# Effect of sports education on students’ classroom motivational climate in physical education: a qualitative investigation

**DOI:** 10.3389/fpsyg.2026.1750258

**Published:** 2026-03-18

**Authors:** Xiubing Zhang, Qiang Zhang, Wenjing Deng

**Affiliations:** 1 Institute of Physical Education and Training, Capital University of Physical Education and Sports, Beijing, China; 2 School of Physical Education, Jinling University, Jinling, China; 3 Chongqing Yong Chuan Vocational Education Central School, Chongqing, China

**Keywords:** engagement, intervention, middle school students, motivational climate, physical education, self-determination theory, semi-structured interviews, sports education

## Abstract

**Background:**

Creating a positive motivational climate in physical education (PE) is crucial for enhancing student engagement. A substantial body of research suggests that the Sports Education Model (SEM) can foster a positive classroom atmosphere. However, there remains a lack of qualitative evidence examining the effects of this model from the students’ experiential perspective.

**Purpose:**

The purpose of this study is to explore the impact of SEM intervention based on basketball on middle school students’ perceptions of the motivational climate in PE classrooms, using qualitative research methods to gain a deeper understanding of its underlying mechanisms.

**Methods:**

The study was conducted over 15 weeks with four first-year middle school classes (*n* = 121) from an eastern province of China. The EG participated in basketball season activities based on the SEM, while the control group received traditional PE instruction. After the intervention, semi-structured interviews were conducted with three students from each group, purposively selected based on varying motivational profiles. Data were analyzed using thematic analysis.

**Results:**

The SEM intervention was associated with positive changes in students’ perceptions of the motivational climate across four key dimensions: mastery climate, relationship support, autonomy support, and performance climate.

**Conclusion:**

SEM is an effective teaching model for fostering an integrated motivational climate that addresses students’ needs for autonomy, relatedness, and competence. This study provides empirical support for the integration of Self-Determination Theory and Achievement Goal Theory in PE practice and offers a structured pathway for teachers to enhance student motivation.

## Introduction

Physical education (PE) plays an irreplaceable role in promoting students’ physical and mental health, cultivating social skills, and shaping positive lifestyles (Liao et al., 2023). However, traditional PE teaching models are often teacher-centered, focusing primarily on repetitive skill practice, which struggles to ignite students’ intrinsic motivation and long-term engagement effectively (Bessa et al., 2021). In this context, the motivational climate—defined as the classroom environment created by the teacher that influences students’ achievement goals and behavior patterns—has been recognized as a key determinant of the success or failure of PE (Ames, 1992).

Self-determination theory (SDT) and achievement goal theory (AGT) provide a solid theoretical foundation for understanding and optimizing classroom motivational climates. SDT asserts that satisfying students’ basic psychological needs for autonomy, relatedness, and competence is central to fostering high-quality intrinsic motivation (Deci and Ryan, 2000). Meanwhile, AGT distinguishes between mastery and performance climates, where the former focuses on personal progress and skill mastery, and the latter emphasizes social comparison and winning (Ames, 1992). Despite their differing perspectives, both theories converge on a common goal: how to create a positive and supportive classroom environment through teaching interventions.

SDT and AGT offer complementary perspectives on student motivation. While SDT explains how social contexts foster intrinsic motivation by supporting basic psychological needs, AGT describes the goal structures individuals adopt in achievement settings. To integrate these insights, this study employs the multidimensional motivational climate framework developed by Soini et al. (2014), which synthesizes elements from both SDT and AGT into a coherent model. This framework is operationalized through a questionnaire that assesses four key dimensions of the classroom climate: mastery climate, performance climate, relationship support, and autonomy support. By providing a validated instrument to capture these interrelated aspects, the Soini et al. (2014) model enables a holistic evaluation of the motivational environment in PE. Consequently, their framework and its four-dimensional structure form the core theoretical and analytical foundation for examining motivational climates in the current study. The Sports Education Model (SEM), as a mature and structured teaching approach, is considered an ideal framework for achieving the aforementioned goals (Siedentop, 1994). SEM incorporates six core features: season-long activities, fixed teams, formal competitions, record keeping, celebratory events, and diverse student roles, all of which aim to provide students with authentic and immersive sports experiences. Theoretically, SEM’s features align closely with the principles of SDT and AGT: student roles and decision-making support autonomy; fixed teams and collaboration foster relatedness; skill development and recognition of diverse abilities satisfy competence needs; and its competitive structure naturally integrates both mastery-oriented and performance-oriented motivational climates.

Although some quantitative studies have confirmed the positive effects of SEM on enhancing student motivation and participation (Giménez-Meseguer et al., 2022; Liao et al., 2023; Nazari et al., 2025), qualitative explorations of how students subjectively experience and perceive the motivational climate created by SEM, as well as the underlying mechanisms, remain insufficient. As emphasized by Santos et al. (2023), “Understanding children’s own perspectives is crucial for designing effective PE.” Therefore, this study adopts a qualitative research approach to explore the following key questions: How do students perceive the motivational climate in PE classrooms during a basketball-based SEM intervention? Through which specific mechanisms does SEM influence their perceptions across the dimensions of mastery climate, relationship support, autonomy support, and performance climate?

By directly presenting students’ voices, this study aims to provide in-depth empirical evidence of the effectiveness of SEM, bridging the gap between theoretical principles and classroom practice. It offers practical insights for PE educators to design and implement more motivating and engaging curricula.

## Methods

### Participants

This study was conducted in a city in eastern China, where the economy is at the mid-level within the province. Participants were recruited from a nine-year integrated school in the city, specifically from four first-year middle school classes taught by the seconder author. A total of 152 students were initially selected (mean age 12.51 ± 1.02 years), all of whom were ethnic Han Chinese. The sample was divided into two groups: the control group (CG), consisting of two classes with 77 students, and the experimental group (EG), consisting of two classes with 75 students. A total of 21 students were excluded from the study due to physical conditions preventing participation in PE classes, failure to complete the survey or physical fitness tests. Consequently, 131 students remained in the final sample for analysis.

To ensure baseline equivalence between the experimental and control groups, a Chi-square test was performed to compare differences in gender and Body Mass Index (BMI) distribution. The results showed no significant differences between the groups in terms of gender (*χ*^2^ = 0.03, *p* = 0.86) and BMI (*χ*^2^ = 0.26, *p* = 0.61), indicating homogeneity between the groups and eliminating potential baseline biases that could affect the experimental outcomes.

### Study design

This study employed a two-phase explanatory sequential mixed-methods design. In the first, quantitative phase, the revised Perceived Locus of Causality in Physical Education scale (PLOC-R; Vlachopoulos et al., 2011) was administered to all participants to assess their baseline levels of autonomous motivation. The results were used to conduct purposeful sampling for the subsequent qualitative phase. In the second phase, semi-structured interviews were carried out to explore students’ subjective experiences and in-depth perceptions of the motivational climate created by different instructional models. This integrated design allowed the quantitative data to guide the selection of information-rich cases, while the qualitative findings provided nuanced insight into the underlying mechanisms shaping students’ classroom experiences.

### Procedures

The teaching for both the experimental and control groups was personally conducted by the researcher—the second author of this study, a PE teacher holding a master’s degree in Sports Pedagogy with 15 years of teaching experience—to maintain consistency in teaching progress, venue equipment, class scheduling, and instructional content across all classes. Before data collection, ethical approval was obtained from the Ethics Review Committee of Capital University of Physical Education and Sports (Approval No.: 2024A085). Permission was also granted by the principal of the intervention school. As the study involved middle school students, informed consent was obtained from both the students and their parents. All participants were reminded that their involvement in the study was entirely voluntary, and their rights to confidentiality, anonymity, and withdrawal from the study at any time were clearly explained. The intervention school was finalized in early 2024, after which a comprehensive and structured intervention plan was developed.

The intervention period lasted for 15 weeks, with a total of 41 class sessions. The first class session involved measuring students’ motivation in PE. At the end of the intervention, in the final class session, semi-structured interviews were conducted with students to assess the effectiveness of the teaching intervention and gain a deeper understanding of their perceptions of the classroom atmosphere. The experimental and control classes each received three 45-min PE lessons per week.

### Sports education

In this study, basketball was selected as the teaching content. Both the experimental and control groups strictly adhered to the “2022 National Curriculum Standards for Physical Education and Health in Compulsory Education” to ensure that the teaching content for both groups was largely consistent.

The basketball intervention based on the SEM for the EG was implemented with high fidelity, and the 15-week teaching was structured into a complete season encompassing pre-season, regular season, and playoffs. Six fixed teams were formed in accordance with the principle of heterogeneity within groups and homogeneity between groups, with the teams balanced in terms of basketball skill levels, gender, and motivational profiles of the students. Each student was assigned 8 specific rotating roles (team captain, coach, referee, scorekeeper, statistician, equipment manager, cheerleader, event organizer), and role rotation was conducted every 3 weeks to ensure that all students experienced different responsibilities. The CG received traditional basketball PE in line with the same curriculum standards, adopting a teacher-centered classroom structure with no fixed teams or role assignments. Students’ progress was evaluated solely based on their performance in weekly skill practice sessions. The intervention details of the EG and the CG are compared in Table 1.

**Table 1:** Comparison of SEM and traditional PE intervention for basketball teaching.

Dimensions	EG (SEM)	CG (Traditional PE)
Team formation	6 fixed teams (6–8 students/team), heterogeneous within groups, homogeneous between groups, balanced skill/gender/motivation	Random temporary groups (4–5 students/team) for drills/scrimmages, no balance criteria
Student roles	8 rotating roles (captain, coach, referee, etc.), role rotation every 3 weeks, all students take diverse roles	No fixed roles; all students are “learners”, and only follow the teacher’s instructions
Season structure	3 stages: Pre-season (Weeks1-6, skill development + team building), Regular season (Weeks7-13, intra-team round-robin competition), Playoffs (Weeks14-15, championship match + award ceremony)	No season structure; single lesson-focused, daily content: warm-up → skill drills → simple scrimmage → cool-down
Task/responsibility rotation	Role rotation every 3 weeks; team members share court setup, warm-up organization, and competition management	No rotation; all tasks (setup, warm-up, drill design) completed by the teacher
Progress evaluation	Comprehensive evaluation (competition performance 40% + role responsibility 30% + skill progress 20% + sportsmanship 10%)	Single evaluation (skill drill performance 100%), only focus on technical proficiency
Culminating event	Formal championship award ceremony (awards for champion team, best referee, most improved player, sportsmanship)	No culminating event; weekly skill check with oral feedback

### Interviews

To gain an in-depth understanding of how students with different motivational levels perceive the motivational climate in PE and their views on participation in physical activity, semi-structured interviews were conducted after the intervention. These interviews also provided multi-dimensional qualitative data to support the study’s findings.

To objectively define and assess students’ motivational characteristics, the entire valid sample (*n* = 131) completed the Perceived Locus of Causality Questionnaire – Revised (PLOC-R; Vlachopoulos et al., 2011) prior to the intervention. This scale consists of 20 items, rated on a 7-point Likert scale (1 = strongly disagree, 7 = strongly agree), measuring five types of motivational regulation (amotivation, external regulation, introjected regulation, identified regulation, and integrated regulation). In this study, the scale demonstrated good internal reliability (Cronbach’s *α* = 0.89 for the overall scale, with subscale α coefficients ranging from 0.78 to 0.91). Based on the scale scores, the Relative Autonomy Index (RAI) was calculated using the formula: RAI = (3 × integrated regulation) + (2 × identified regulation) – introjected regulation – (2 × external regulation) – (3 × amotivation). Students were then divided into high, medium, and low levels of autonomous motivation based on the tertiles of their RAI scores (the top 33% were classified as high motivation, the middle 33% as medium motivation, and the bottom 33% as low motivation).

Following the intervention and based on the quantitative grouping results above, the researcher selected six students for semi-structured interviews—three from the EG and three from the CG (with equal gender representation, as detailed in Table 2). Following the methodology of Knowles et al. (2018) to ensure a rich cross-case analysis, students with varying levels of motivation in PE were selected. The specific purposive sampling procedure was as follows: (1) The experimental and control groups were stratified according to RAI motivational characteristics and gender. (2) One student was randomly selected from each stratum (high/medium/low motivation, gender-balanced) within both groups, resulting in a total of 6 students (3 per group). (3) Selected students and their parents were contacted to obtain informed consent for the one-on-one semi-structured interviews. (4) Non-participating students were replaced with other students from the same stratum to ensure sample representativeness. The aim of this small-sample, in-depth interview approach was to capture the richness, depth, and typicality of experiential data, rather than to achieve statistical generalization. Using pre-test PLOC-R scores to assess autonomous motivation, students representing high, medium, and low levels of self-determined motivation were purposively selected from each group, ensuring diversity and representativeness in the qualitative data.

**Table 2:** Interview participant information (*n* = 6).

Participant	Class	Gender	RAI	Level
P 1	EG	Male	9.33	High
P 2	EG	Female	4.5	Medium
P 3	EG	Male	1.33	Low
P 4	CG	Female	10.16	High
P 5	CG	Male	6.67	Medium
P 6	CG	Female	2.83	Low

The interview questions were strictly designed with reference to the interview outline by Vazou et al. (2005), centering on the four-dimensional motivational climate framework and a comprehensive perception dimension. For example, the mastery climate dimension included the question *Does your PE teacher often provide you with positive feedback and encouragement during lessons?*; the relationship support dimension included *Do you get along well with your classmates and teacher?*; the autonomy support dimension included *Do you think the PE teacher has given you and your classmates enough opportunities to make choices during lessons?* And do *you feel you can do things in your own way?*; the performance climate dimension included *Can you describe the atmosphere in your team during training and competitions? Could you elaborate more on it? Why do you think this kind of atmosphere exists? How do you view the atmosphere of comparison and competition within your team?* For details, see the Appendix S1. All interviews were conducted one-on-one in a quiet classroom and audio-recorded with the participants’ consent, with each interview lasting 15 to 20 min. During the interviews, standardized questioning was strictly conducted in accordance with the above outline to ensure consistent questioning methods and question sequences for all interviewees. Only natural follow-up questions were asked based on the interviewees’ responses to explore in-depth information, without adding extra questions. Verbatim transcription of the interview content was completed within 48 h after the interviews, and all personal identifying information was removed from the transcripts to ensure anonymity. Finally, the transcribed texts were ultimately sorted, managed and coded by the aforementioned motivational climate dimensions. To avoid interviewer bias, a neutral PE teacher of the same grade who was not involved in the intervention teaching served as the interviewer; the teacher received special training on the interview protocol and ethical guidelines in advance, and clearly informed the students before the interviews that the content would be kept strictly confidential and would not affect their PE academic evaluation. This was done to enhance the participants’ sense of security and ensure the authenticity of the feedback information across all dimensions.

### Data analysis

Quantitative data, including students’ baseline autonomous motivation scores (revised Perceived Locus of Causality in Physical Education Scale) and Relative Autonomy Index, were collated and analyzed using SPSS 24.0. Descriptive statistics (mean ± standard deviation) and chi-square tests were used to verify the baseline equivalence between the EG and the CG. Qualitative interview data were systematically analyzed using the six-phase thematic analysis method proposed by Braun and Clarke (2006), with the four-dimensional motivational climate framework, combined with the comprehensive perception dimension, as the core analytical thread. The analysis followed the sequential steps of familiarization with the transcripts, generation of initial descriptive codes by dimension, searching and collating potential themes based on each dimension, reviewing and revising themes to ensure their internal consistency and external distinctiveness, clearly defining and naming the core themes and sub-themes under each dimension, and compiling research findings combined with interview excerpts.

To ensure the rigor, reliability, and validity of qualitative analysis (Braun and Clarke, 2006), the following strategies were implemented in this study: (1) Blinded coding and reliability test: The first and second authors were blinded to group information and independently coded 30% of the interview data by dimension. The inter-coder reliability was tested via Cohen’s Kappa coefficient, with the result showing *κ* = 0.87 (*p* < 0.001), indicating excellent consistency. All coding discrepancies were resolved through group discussion to reach a consensus. (2) Triangulation validation: Triangulation validation was conducted on the interview data of students with different motivational levels (high, medium, low) and genders by dimension to examine the similarities and differences in their perceptual perspectives. (3) Member checking: The core research results and transcript excerpts of each dimension were fed back to 6 interviewees to confirm the accuracy of data interpretation. (4) Audit trail: The entire analysis process, including coding, theme construction, and revision was fully documented by dimension to ensure research transparency. (5) Reflexivity statement: The research team completed a reflexivity statement before analysis, documenting preconceptions and potential biases regarding the impacts of the Sport Education Model on each dimension to reduce subjective interference. In addition, a large number of anonymized original interview quotations were cited by motivational climate dimension in the presentation of research results.

## Results

Based on semi-structured interviews with 6 research participants (3 in the EG and 3 in the CG) with different motivation levels, this study explored the impact of teaching intervention on students’ perceptions of different motivational climate dimensions in PE classes. The thematic analysis method was adopted to code and analyze the interview texts, and four core themes (mastery climate, relatedness support, autonomy support, and performance climate) were extracted, along with eight sub-themes: *targeted positive feedback, professional role recognition, peer mutual assistance and cooperation, equal and frequent teacher-student interaction, autonomous choice and decision-making participation, role autonomous assignment experience, positive competition and focus on progress, and negative competition and focus on winning or losing.* (see Table 3).

**Table 3:** Summary of core themes, sub-themes and example quotation coding.

Core themes	Sub-themes	Group-motivation Level	Example quotations (code: research participant)
Mastery climate	Targeted positive feedback	EG-High	“In the high and low dribbling practice, the teacher praised me, saying ‘This movement is quite standard’, and asked other students to imitate my movement.” (P1)
	Professional role recognition	EG-Low	“The teacher said I was the best referee among us, and this recognition made me feel valuable in the class.” (P3)
Relatedness support	Peer mutual assistance and cooperation	EG-Medium	“I cannot always make accurate shots, and my teammates will take the initiative to teach me the force method; I also help my teammates correct their dribbling movements.” (P2)
	Equal and Frequent Teacher-Student Interaction	EG-High	“The teacher often joins our discussions, asks about the problems we encounter in team practice, and works with us to find solutions.” (P1)
Autonomy Support	Autonomous choice and decision-making	EG-High	“I suggested leaving more time for games at the beginning of the class, and the teacher adopted my suggestion. Everyone practiced happily that day.” (P1)
	Role autonomous assignment experience	EG-Low	“The teacher asked me to be a referee, let us ‘referees’ learn the rules at home, and I was responsible for blowing the whistle during class games. I found it very interesting.” (P3)
Performance Climate	Positive competition and focus on progress	EG-Medium	“This atmosphere is both competitive and supportive. Everyone is trying to improve themselves and will not overly focus on whether they surpass their teammates.” (P2)
	Negative competition and focus on winning or losing	CG-High	“In the class games, everyone only wants to win. If they lose, they will blame each other, and the teacher will not guide us to correctly view winning or losing.” (P4)

### Mastery climate

#### Targeted positive feedback

Targeted positive feedback refers to teachers’ specific affirmation of students’ movements, performances, or progress in PE classes, which is different from general and vague praise and can effectively make students perceive their own abilities and efforts. This sub-theme is mainly reflected in the EG, and there are differences in perception among students with different motivation levels.

P1 (EG, male, RAI = 9.33) with a high motivation level had higher requirements for the professionalism and specificity of teachers’ feedback, and could clearly perceive the targeted nature of positive feedback. He mentioned in the interview: “I can feel the recognition brought by the teacher’s praise, especially when I perform well. For example, in the high and low dribbling practice, the teacher praised me, saying ‘This movement is quite standard’, and asked other students to imitate my movement. This made me feel that my efforts were seen, and my class participation became more active—not only for my own progress, but also to let the teacher see that I was working hard.” This specific feedback accurately met his pursuit of competence and further strengthened his learning initiative.

For P3 (EG, male, RAI = 1.33) with low motivation level, targeted positive feedback was more reflected in the teacher’s tolerant encouragement. He said: “The teacher encouraged us to try boldly and often said ‘Don’t be afraid of making mistakes’, which made me no longer afraid to participate in the class.” This kind of targeted encouragement aimed at reducing his participation fear effectively helped him establish initial learning confidence. In contrast, students in the CG rarely received targeted positive feedback, mostly only vague praise such as “Well done,” which made it difficult for them to perceive their own progress and abilities.

#### Professional role recognition

Professional role recognition refers to teachers’ affirmation of students’ performance in the specific professional roles assigned in PE classes (such as referee, safety officer, vice-captain), which is an important way for students to perceive their own value and enhance their sense of competence, and is mainly reflected in the EG. P3 (EG, male, RAI = 1.33) with a low motivation level gained a strong sense of value through professional role recognition. He mentioned: “This semester, the teacher also asked me to be a referee, let us ‘referees’ learn the rules at home, and I was responsible for blowing the whistle for our team during class games. I found it very interesting… The teacher also said that I was the best referee among us.” This recognition of his role as a referee made him get rid of the sense of inferiority in PE classes and feel that he had unique value in the class.

P2 (EG, female, RAI = 4.50) with a medium motivation level also perceived professional role recognition in her role as a group safety officer: “As the group safety officer, I reminded everyone to pay attention to movement standards, and the teacher praised me for being responsible. This recognition also made me more motivated.” For students in the EG, regardless of their motivation level, professional role recognition became an important means to perceive the mastery climate, while students in the CG had no such role experience and thus could not obtain corresponding recognition.

### Relatedness support

#### Peer mutual assistance and cooperation

Peer mutual assistance and cooperation refer to the positive interaction between students in PE classes, including helping each other correct movements, sharing skills, and cooperating to complete tasks, which is an important manifestation of students’ perception of group warmth and mutual support, and is mainly reflected in the EG with a fixed team mechanism. P2 (EG, female, RAI = 4.50) with a medium motivation level had the most intuitive perception of peer mutual assistance and cooperation: “Very good, the relationship in our team is very harmonious. For example, I cannot always make accurate shots, and my teammates will take the initiative to teach me the force method; I also help my teammates correct their dribbling movements. Everyone makes progress together, feeling very warm.” This two-way mutual assistance not only improved her movement skills but also made her feel the support from peers.

P1 (EG, male, RAI = 9.33) with a high motivation level also recognized the role of peer mutual assistance in improving learning efficiency: “During group practice, we supervise and correct each other, which is much more effective than practicing alone.” In the CG, due to the lack of a fixed team mechanism, students were mainly focused on their own practice, and there was almost no active peer mutual assistance and cooperation. P4 (CG, female, RAI = 10.16) with a high motivation level said: “Most of the time we just practice movements and rarely talk. Everyone is only focused on their own practice and will not take the initiative to help others.”

#### Equal and frequent teacher-student interaction

Equal and frequent teacher-student interaction refers to teachers’ initiative to communicate with students in PE classes, listen to their opinions, answer their questions, and participate in their team activities, showing an equal and cooperative attitude, which is mainly reflected in the EG.

P1 (EG, male, RAI = 9.33) with a high motivation level described this kind of interaction in detail: “The teacher also often joins our discussions, asks about the problems we encounter in team practice, and works with us to find solutions. It feels like the teacher is not a superior instructor, but more like our teammate.” This equal interaction made him feel respected and enhanced his sense of belonging in the class. P2 (EG, female, RAI = 4.50) with medium motivation level also felt the warmth brought by frequent teacher-student interaction: “The teacher will take the initiative to ask about my practice experience and patiently answer my questions about movements, without a sense of distance.” In contrast, teacher-student interaction in the CG was mainly one-way guidance. P6 (CG, female, RAI = 2.83) with a low motivation level said: “I practice hard, but I have little interaction with classmates and dare not ask the teacher questions.” This lack of equal and frequent interaction made students in the CG feel a sense of distance from teachers.

### Autonomy support

#### Autonomous choice and decision-making

Autonomous choice and decision-making refers to students’ opportunity to independently choose practice content, practice methods, or participate in class decision-making in PE classes, and teachers respect and adopt their reasonable suggestions, which is a key sub-theme reflecting autonomy support and is mainly reflected in the EG. P1 (EG, male, RAI = 9.33) with a high motivation level had strong autonomous awareness and actively participated in class decision-making: “Yes, the teacher will provide different learning methods according to our interests and abilities, giving us room for choice. For example, when grouping, the teacher originally referred to our previous test scores, but some students were not satisfied with the grouping and put forward their own opinions. The teacher carefully listened to our feedback and made adjustments… Once, I suggested not spending too much time practicing dribbling at the beginning of the class, but leaving more time for games. The teacher adopted my suggestion, and everyone practiced happily that day and was more motivated.”

P2 (EG, female, RAI = 4.50) with medium motivation level, although not active in putting forward opinions, also enjoyed the right of autonomous choice: “The teacher will let us choose the practice content ourselves. For example, after the basic class, we can choose to practice shooting, dribbling, or play small games with teammates. I usually choose to play games with teammates because it is more interesting and can exercise our cooperation ability.” Students in the CG had almost no opportunity for autonomous choice and decision-making. P4 (CG, female, RAI = 10.16) said: “The teacher rarely gives us choices. We all practice movements according to his arrangement: jogging first, then practicing dribbling and passing, and finally free practice. The time for free practice is also very short, and there is no time to do what we want.”

#### Role autonomous assignment experience

Role autonomous assignment experience refers to students’ opportunity to obtain and experience specific professional roles (such as referee, captain, coach) assigned by teachers in PE classes, and have a certain degree of autonomy in role performance, which is another important sub-theme reflecting autonomy support and is unique to the EG.

P3 (EG, male, RAI = 1.33) with low motivation level gained a sense of autonomy and fulfillment through the role of referee: “The teacher asked me to be a referee, which is something I have never done before. He let us ‘referees’ learn the rules at home, and I was responsible for blowing the whistle and judging fouls during class games. I found it very interesting and fulfilling. The teacher also asked us about our feelings as referees, whether there were any difficulties, and would adjust the game rhythm according to our feedback, making me feel that my ideas were valued.” P1 (EG, male, RAI = 9.33) with a high motivation level also had a rich role autonomous assignment experience as the vice-captain: “As the vice-captain, I can participate in formulating the group training plan, and my teammates will listen carefully to my arrangements.” This role experience not only gave students the right of autonomy but also enhanced their sense of responsibility and participation. Students in the CG had no such role assignment, so they could not obtain corresponding autonomous experience.

### Performance climate

#### Positive competition and focus on progress

Positive competition and focus on progress refer to the competitive atmosphere in PE classes that focuses on personal progress and team cooperation, where peers encourage each other, and teachers guide students to view competition correctly, with the core of “competing with oneself” rather than “competing with others”, which is mainly reflected in the EG. P2 (EG, female, RAI = 4.50) with a medium motivation level described this competitive atmosphere in detail: “The atmosphere in our team is very good, and sometimes teammates will remind each other. For example, if some movements are not in place during training, teammates will say ‘You should do this’ and then give encouragement, instead of laughing at me for not doing well. I think this atmosphere is both competitive and supportive. In general, everyone is trying to improve themselves… Although I will not overly focus on whether I surpass my teammates, when they perform well, I will feel that I should also work harder. This comparison gives me the motivation of ‘not wanting to fall behind’.”

P3 (EG, male, RAI = 1.33) with a low motivation level, who was originally afraid of competition, gradually overcame his fear under this positive atmosphere: “In our team, everyone will not blame anyone for not playing well. Teammates will encourage me, and the teacher will also say, ‘As long as you try your best, making progress than last time is winning’. Slowly, I am no longer afraid of competition and am willing to take the initiative to participate in games. Even if I don’t perform well, I will try my best.” P1 (EG, male, RAI = 9.33) also pointed out that this positive competition promoted the overall progress of the team: “During training, we will compete to see who is more proficient in dribbling and has a higher shooting hit rate, but it is not that kind of malicious competition. Instead, we will share skills and help each other progress. During the game, we pay more attention to team cooperation rather than simply winning or losing.”

#### Negative competition and focus on winning or losing

Negative competition and focus on winning or losing refer to the competitive atmosphere in PE classes that focuses on game results and personal gains and losses, where peers blame each other for failure, and teachers lack correct guidance, which is mainly reflected in the CG.

P4 (CG, female, RAI = 10.16) with a high motivation level was dissatisfied with this negative competitive atmosphere: “In the class games, everyone only wants to win. If they lose, they will blame each other, saying who played badly and dragged the team down, and even quarrel. The teacher will not guide us, just stand aside and watch. The team that wins the game will be praised by the teacher, and the team that loses will be criticized by the teacher for ‘not working hard enough’. This kind of competition is very oppressive. Sometimes I obviously play well, but because the team loses, I will still be blamed by my teammates. Slowly, I don’t want to participate in the game anymore.”

P5 (CG, male, RAI = 6.67) with a medium motivation level had a negative attitude towards competition due to this atmosphere: “I don’t like the competitive atmosphere in PE classes. Everyone is too focused on winning or losing. If I don’t perform well, I will be laughed at by my teammates, and the teacher will not encourage me, only saying, ‘Why are you so stupid, you can’t even do such a simple movement well’. So every time there is a game, I try to hide behind, not take the initiative to participate in offense and defense, as long as I don’t make mistakes, there is no motivation at all.” P6 (CG, female, RAI = 2.83) even completely avoided competition: “I dare not participate in games because I am not good at playing. I am afraid of being blamed by everyone if I lose, and the teacher will not care about my feelings… After losing the game, my teammates all laughed at me as a ‘burden’, and the teacher also criticized me for ‘not practicing earnestly’. Since then, I have been more afraid of games.”

## Discussion

This qualitative study explored the impact of a SEM teaching intervention on seventh-grade students’ perceptions of the motivational climate in PE. Grounded in SDT and AGT, and organised around four core dimensions and their eight sub-themes, the discussion interprets the findings, places them in dialogue with existing literature, acknowledges limitations, and outlines practical implications for PE reform at the middle-school level.

### SEM satisfies basic psychological needs: an SDT-based interpretation

From an SDT perspective (Ryan and Deci, 2000), the SEM intervention systematically satisfied students’ three basic psychological needs—autonomy, competence, and relatedness—through its structured pedagogical mechanisms, thereby fostering positive perceptions of the motivational climate.

The satisfaction of *autonomy* was reflected in the sub-themes “autonomous choice and decision-making” and “experience of autonomous role assignment”. By assigning students diverse roles (e.g., referee, captain) and involving them in classroom decisions, SEM returned the locus of learning to the students. As described by the highly motivated student P1 (participation in group-formation decisions) and the low-motivation student P3 (serving as a referee and independently fulfilling role responsibilities), these experiences gave students a sense of self-determination in their actions, effectively stimulating their intrinsic motivation for classroom engagement. In contrast, the traditional teacher-dominated, student-executed model of the CG kept students in a passive-receptive state with little room for autonomous choice—a situation consistent with Deci and Ryan (2000) assertion that lack of autonomy support undermines intrinsic motivation. Our findings further suggest that SEM, through “role empowerment” and “decision-making delegation”, can effectively break the autonomy dilemma typical of traditional PE classes.

The fulfilment of the *competence* need was manifested in the sub-themes “targeted positive feedback” and “professional role recognition”. SDT posits that competence is built on specific, positive efficacy feedback received from the environment. In the EG, teachers provided concrete praise (e.g., “your movement is quite standard”) and role-based recognition (e.g., “best referee” awarded to P3), enabling students of varying motivational levels to clearly perceive their own progress and value. This was especially crucial for low-motivation students, helping them overcome learned helplessness and rebuild confidence in PE (Xiang and Lee, 2002). When viewed through AGT (Ames, 1992), SEM clearly fostered a mastery-oriented climate: students were encouraged to use their own previous performance as a reference point rather than engaging in social comparison. By internalising competence evaluation criteria as personal improvement, this climate translates the abstract concept of competence support into concrete classroom practice. The CG’s use of vague, generic praise failed to convey clear efficacy signals and therefore could not satisfy students’ competence needs.

Notably, SEM’s multifaceted recognition system created an inclusive mastery climate: teachers not only attended to skill acquisition but also offered personalised feedback (e.g., “standard movement”) and acknowledged role-specific expertise (e.g., “best referee”), ensuring that every student could experience a sense of achievement in his or her area of strength. This “personalised recognition strategy” aligns closely with the findings of Xiang and Lee (2002).

The satisfaction of the *relatedness* need emerged from the sub-themes “peer cooperation and mutual help” and “equal and frequent teacher–student interaction”. SEM’s “stable team mechanism” and “cooperative task design” provided students with sustained, deep opportunities for social interaction. EG students described peer support (e.g., P2 and teammates correcting each other’s movements) and teacher involvement (e.g., P1’s account of the teacher joining discussions to solve problems together), reflecting positive peer relationships and emotional bonds with the teacher. Such interactions enabled students to feel a sense of belonging and emotional support in the classroom, thereby enhancing their positive perceptions of the motivational climate. SDT emphasises that relatedness satisfaction is a fertile ground for intrinsic motivation; SEM’s structural design of a supportive social network provides an ideal context for fulfilling this need. The CG, lacking a stable team structure, experienced sparse peer interaction and predominantly one-way, command-style teacher communication, resulting in feelings of alienation and weak group belonging—further corroborating the critical role of social interaction in motivational processes.

### The dialectical relationship between mastery and performance climate: an AGT-based extension

AGT distinguishes two main dimensions of motivational climate: mastery climate and performance climate (Ames, 1992). Our study further suggests that these dimensions can be seen as *contextualised manifestations of SDT’s “competence support”*: mastery climate represents the positive pathway for competence support, whereas performance climate represents its absence or distortion.

The SEM in the EG transformed competitive pressure into motivation for improvement, shaping a *healthy perception of performance climate*. Student P2’s remark—“I don’t care too much about surpassing my teammates, but I feel I should work harder”—reflects a distinctive competitive mindset: one that values personal achievement while avoiding the anxiety of social comparison. This mindset emerged because SEM *reframed competition* through two key mechanisms: (a) grouping ensured intra-team diversity and inter-team homogeneity, and (b) the evaluation system considered both outcomes and processes, turning competition from a “zero-sum game” into a “catalyst for shared progress”. Rodrigues et al. (2020) noted that a student-led performance climate enhances autonomy and competence, whereas an excessively teacher-dominated performance climate can harm relatedness. By empowering students to participate in the enforcement of competition rules and in evaluation, SEM achieved the former.

In contrast, the CG exhibited a *distorted performance climate*: students were overly concerned with winning or losing; winners were praised, losers were blamed, and teammates blamed each other for failures; low-motivation students avoided competition for fear of failure. In such a climate, feelings of competence depend entirely on results, while individual effort and improvement are ignored—SDT’s competence support is completely absent. This finding reinforces the notion that only a mastery climate centred on self-improvement can truly satisfy the competence need; a performance climate that is not properly guided becomes a “suppressor” of that need.

### SEM’S unique contributions in dialogue with existing research

The results of this study resonate with previous national and international research on SEM and motivational climate, while also making several unique contributions. Internationally, Siedentop (1994) seminal work on SEM emphasised its capacity to enhance classroom engagement through role assignment and teamwork; Hastie et al.’s (2014) quantitative study confirmed that SEM effectively creates a mastery-oriented climate. Domestically, Li (2018) reported that SEM interventions improved students’ perceptions of competence and relatedness, thereby enhancing the motivational climate. Our study not only corroborates these findings but also extends the literature in the following ways.

#### First, we offer a finer-grained dimensional analysis

While most existing studies have examined SEM’s impact on overall motivational climate, our qualitative analysis extracted eight specific sub-themes, revealing the internal structure of the four core dimensions (mastery climate, performance climate, autonomy support, relatedness support). This transparency clarifies the mechanisms through which SEM operates.

#### Second, we reveal heterogeneity of effects

By considering students with different initial motivation levels, we found that highly motivated students benefited more from autonomous decision-making and targeted feedback, whereas low-motivation students gained confidence primarily through role recognition and tolerant encouragement. This suggests that SEM’s effects are not uniform; its multifaceted design can address the heterogeneous needs of a diverse student body.

#### Third, we extend the evidence base to a younger age group and a specific sport context

Focusing on basketball in the seventh grade—a developmental stage marked by increasing sensitivity to peer comparison and social evaluation—our study deepens the understanding of how SEM functions in early adolescence, an age group that has received relatively limited attention in the SEM literature. By integrating findings with recent studies such as Rodrigues et al. (2020) and Weiss and Smith (2002), we provide a more nuanced picture of SEM’s motivational mechanisms in middle-school PE.

## Limitations

This study provides in-depth qualitative evidence for understanding the influence of the SEM on the motivational climate in PE classrooms. However, several constraints must be acknowledged when interpreting the results. The intervention was delivered by a single researcher within one school, using a convenience sample of limited size. While this aided implementation fidelity, it may restrict the generalizability of findings across different instructors, schools, and regional settings. In addition, although the traditional instruction program used for the CG followed curriculum guidelines, it was not a rigorously validated standardized protocol, which could introduce uncontrolled variables into the comparative analysis.

At the methodological level, while interview data from students with high, medium, and low motivation levels offer rich perspectives, the sample size remains small, and the analysis primarily relies on thematic identification by the research team, which may still carry subjective risks. Additionally, the study did not systematically monitor whether the teacher unconsciously applied additional motivational strategies beyond the structural features of the instructional models during the teaching process, which could represent a potential confounding factor.

### Practical implications and future research directions

Building on the findings of this study, an integrated perspective on practical applications and future research is proposed. Teachers should consciously adopt clearly structured, student-centered pedagogical models such as SEM. Its defining features—persistent teams, diverse roles, and a season format—naturally foster an environment supportive of autonomy, competence, and relatedness. In practice, teachers should prioritize providing personalized, process-oriented feedback and offering students meaningful choices within the instructional framework, such as in tactical decisions or role responsibilities. This approach directly contributes to cultivating a mastery-oriented classroom culture.

Educational institutions and departments must shift the focus of teacher professional development from purely technical skill training to comprehensive training that includes evidence-based pedagogies like SEM, coupled with an understanding of their underlying motivational theories. Concurrently, reforming teaching evaluation systems to incorporate dimensions such as student motivation, engagement, and social skills is essential to guide instructional practice toward promoting holistic student development. Such systemic support is crucial for the effective dissemination and sustained implementation of innovative teaching methodologies.

Future research should conduct cluster-randomized controlled trials involving multiple teachers and schools, utilizing validated instructional programs to strengthen causal inference and generalizability. The moderating role of variables such as student gender and initial motivation levels could be explored. Employing more robust longitudinal mixed-methods designs would allow for the quantification of long-term effects and the investigation of the transfer of motivation and skills. Furthermore, in-depth research into teachers’ beliefs, challenges, and support needs when implementing new pedagogies is necessary to enhance the understanding of classroom motivational climates from the instructional perspective.

## Data Availability

The original contributions presented in the study are included in the article/Supplementary material, further inquiries can be directed to the corresponding author.
