# Supplementary material for: Effect of sports education on students’ classroom motivational climate in physical education: a qualitative investigation
Source: Front Psychol. 2026 Mar 18;17:1750258. doi: 10.3389/fpsyg.2026.1750258 (PMC13038968; doi:10.3389/fpsyg.2026.1750258)
Supplement: Supplementary file 1 [file Data_Sheet_1.pdf]

Supplementary file 1

Does your physical education teacher often provide you with positive feedback and encouragement during lessons?
a. Yes. How do you think the teacher's encouragement makes a difference? Do you believe the teacher’s feedback and encouragement have a positive impact on your learning? Why?
b. No. Would you like to receive encouragement from the teacher? Why?

Do you get along well with your classmates and teacher?
a. Yes. In your team (or class), how do you feel about expressing your opinions? How do you see your role in the team (or class)?
b. No. Why?

Do you think the physical education teacher has given you and your classmates enough opportunities to make choices during lessons?
a. Yes. How has the teacher provided these opportunities?
b. No. Does the lack of opportunities affect your participation and initiative in physical activities?

Do you feel you can do things in your own way?
a. Yes. Can you describe how you have been involved in decision-making?
b. No. Why do you feel the classroom doesn’t offer freedom?

Can you describe the atmosphere in your team during training and competitions? Could you elaborate more on it? Why do you think this kind of atmosphere exists? How do you view the atmosphere of comparison and competition within your team?

Do you feel more actively engaged in activities during physical education classes compared to before?
a. Yes. Why do you think you’ve become more engaged?
b. No. Why do you not participate as actively in the class activities?

How do you feel emotionally compared to previous physical education classes this semester?

Do you think the content you’ve learned in physical education classes has become more valuable or practical? Are you more willing to actively think and apply the skills learned during physical education?
a. Yes. Could you elaborate on that?
b. No. Why?
